# Editorial: The role of non-coding RNA in the diagnosis and treatment of infection induced gastrointestinal cancers

**DOI:** 10.3389/fmed.2023.1153906

**Published:** 2023-02-17

**Authors:** Qian Xu, Jianping Liu

**Affiliations:** ^1^Tumor Etiology and Screening Department of Cancer Institute and General Surgery, The First Affiliated Hospital of China Medical University, Shenyang, China; ^2^Department of Gastroenterology, The First Affiliated Hospital of Nanchang University, Nanchang, Jiangxi, China

**Keywords:** non-coding RNA, infection, cancer, diagnosis, treatment

The selected articles address the problems associated with the microbial infections leading to tumor and elaborated the roles of involved non-coding RNAs from different perspectives. Microbial infections lead to long-term chronic inflammation, metabolic homeostasis disorders, and immune regulation, which communally contributes to the occurrence of digestive system tumors. It has been well-recognized that *Helicobacter pylori* (*H. pylori*) infection can cause systemic multiple organ diseases and even tumors, such as gastric cancer (GC) (1). Other pathogenic microorganisms, including Epstein-Barr virus (EBV) and various hepatitis viruses, are all related to the occurrence of gastrointestinal cancer, liver cancer, and other digestive system cancers (2, 3). Non-coding RNAs, including miRNA, lncRNA, circRNA, piRNA, etc., can regulate gene expression at epigenetic, transcriptional, and post-transcriptional levels, through methylation or histone modification, chromatin remodeling, or competitive inhibition. Non-coding RNAs can be used as molecular markers for diagnosis, prognosis prediction, and treatment of digestive system tumors caused by microbial infections.

In this Research Topic, Long et al. identified the stage-associated exosome miRNAs in colorectal cancer by improved robust and corroborative approach embedded miRNA-target network, and three miRNAs (miR-23b-3p, miR-301a-3p, and miR-194-3p) were demonstrated to be the most stably expressed stage-associated miRNAs in CRC serum exosomes, cell exosomes and tissues, suggesting that these exosome miRNAs could be potential biomarkers for colorectal cancer. Not only miRNAs, the circulating Epstein-Barr virus DNA could also be a diagnostic and prognostic biomarker for the gastrointestinal tumor. He et al. further showed that circulating EBV DNA might play a predictive role in distinguishing EBVaGC from EBVnGC (AUC 0.79, *P* < 0.001), and was associated closely with better overall survival (HR 0.45, *P* = 0.039). EBV infection in patients with gastric cancer may be linked to hepatic impairment and immune response. Circulating cell-free EBV DNA is not only a biomarker for the screening of an EBV-related GC subtype, but also an independent prognosis factor for the long-term survival benefit in GC patients. Next, Li et al. described some mechanisms about the infection of hepatitis B virus (HBV). They found that a clathrin-binding membrane protein epsin3 (EPN3) could negatively regulate the expression of HBV RNA. EPN3 expression was induced by transfection of an HBV replicon plasmid, while the HBV-RNA level was reduced in hepatic cell lines and murine livers hydrodynamically injected with the HBV replicon plasmid. Viral RNA reduction by EPN3 was dependent on transcription, and independent of epsilon structure of viral RNA. Viral RNA reduction by overexpression of p53 or IFN-α treatment, was attenuated by knockdown of EPN3, suggesting its role downstream of IFN-α and p53. Taken together, their studies demonstrated the anti-HBV role of EPN3.

In addition, there were two reviews summarizing the roles of non-coding RNAs in the microbial infections related tumors in this Research Topic. Yan et al. summarized the roles of Non-coding RNA in hepatitis B virus-related hepatocellular carcinoma (HCC) using a bibliometric analysis method, and Liu et al. reviewed the roles of non-coding RNA in the diagnosis and treatment of *Helicobacter pylori*-related GC, with a focus on inflammation and immune response. The former review analyzed the top 100 keywords using a co-occurrence cluster method and found four clusters which were the main hot topic for the association between non-coding RNAs and HBV-related HCC: (1) non-coding RNA as a molecular marker for the diagnosis and prognosis of HBV-related HCC; (2) dysregulation of non-coding RNA by HBV X protein (HBx); (3) effects of non-coding RNA on the biological behaviors of HBV-related HCC; and (4) epidemiological study for the effects of non-coding RNA on the risk of HBV-related HCC. The latter one briefly described the effects of non-coding RNAs on *H. pylori*-related GC from the perspective of inflammation and immune response, as well as their associations with inflammatory reactions and immune microenvironment. They outlined the potential of ncRNAs as markers for the early diagnosis, prognosis, and treatment of *H. pylori*-related GC. The ncRNAs involved in *H. pylori*-related GC may hold great promise as novel therapeutic targets for immunotherapy.

The Research Topic “*The role of non-coding RNA in the diagnosis and treatment of infection induced digestive system cancers*” represents an important contribution to the regulatory mechanisms of non-coding RNAs in the complex molecular network of digestive system tumors caused by microbial infections, reveals current research gaps, and finally stimulates new ideas for future researches on the topic.

## Author contributions

QX wrote the article. JL revised the manuscript. All authors shared and approved the contents.
